# Argininosuccinate synthase 1, arginine deprivation therapy and cancer management

**DOI:** 10.3389/fphar.2022.935553

**Published:** 2022-07-15

**Authors:** Naihui Sun, Xing Zhao

**Affiliations:** ^1^ Department of Anesthesiology, The First Affiliated Hospital of China Medical University, Shenyang, China; ^2^ Department of Pediatrics, The First Affiliated Hospital of China Medical University, Shenyang, China

**Keywords:** metabolic reprogramming, arginine, amino acid, resistance, prognosis

## Abstract

Metabolic reprogramming is an emerging hallmark of tumor cells. In order to survive in the nutrient-deprived environment, tumor cells rewire their metabolic phenotype to provide sufficient energy and build biomass to sustain their transformed state and promote malignant behaviors. Amino acids are the main compositions of protein, which provide key intermediate substrates for the activation of signaling pathways. Considering that cells can synthesize arginine *via* argininosuccinate synthase 1 (ASS1), arginine is regarded as a non-essential amino acid, making arginine depletion as a promising therapeutic strategy for ASS1-silencing tumors. In this review, we summarize the current knowledge of expression pattern of ASS1 and related signaling pathways in cancer and its potential role as a novel therapeutic target in cancer. Besides, we outline how ASS1 affects metabolic regulation and tumor progression and further discuss the role of ASS1 in arginine deprivation therapy. Finally, we review approaches to target ASS1 for cancer therapies.

## Introduction

**FIGURE 1:**
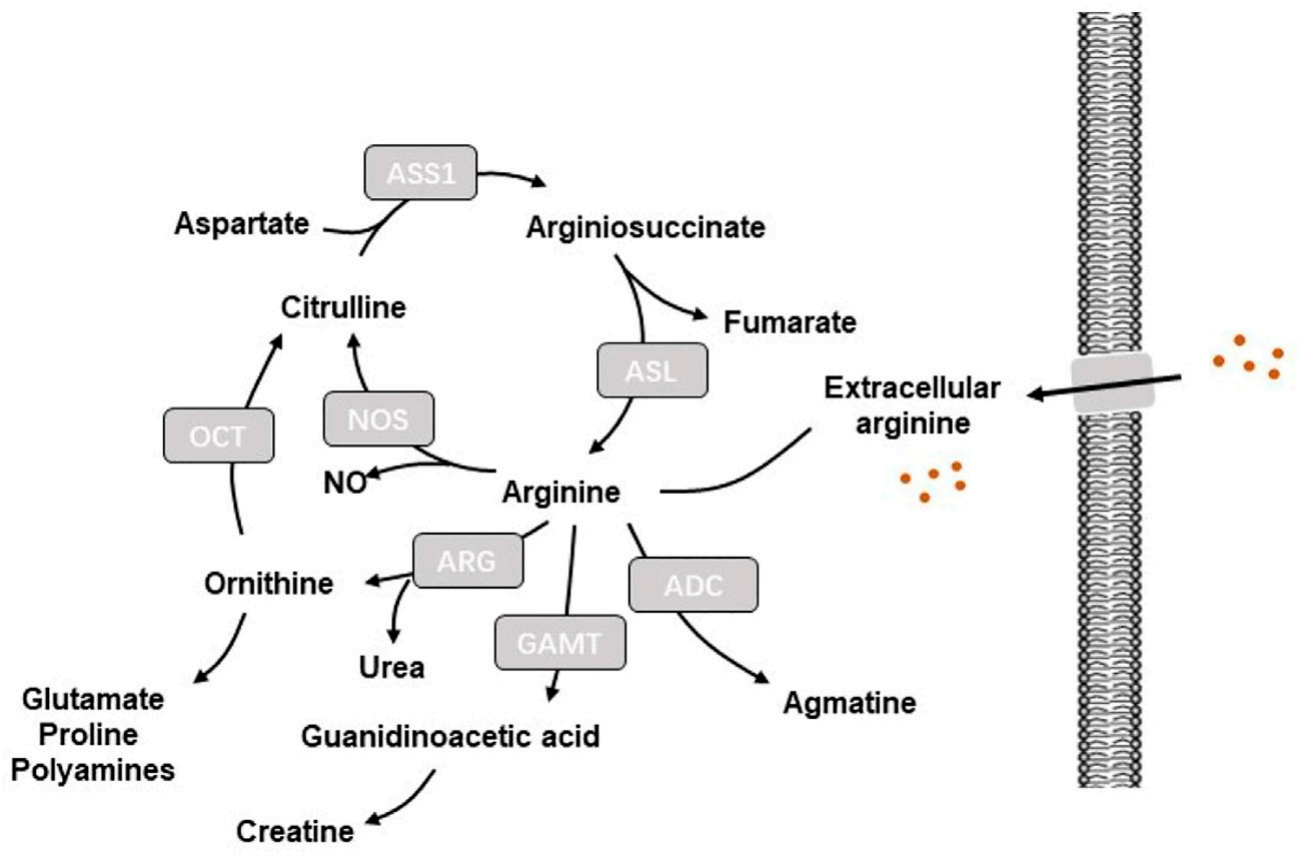
Illustration of arginine metabolism in cells. ADC, arginine decarboxylase; ADI, arginine deiminase; ARG, arginase; ASL, arginine-succinate lyase; ASS1, arginine-succinate synthetase 1; GAMT, guanidinoacetate-N-methyltransferase; NO: nitric oxide; NOS: nitric oxide synthase; OCT, Ornithine carbamoyl transferase.

A key characteristics of tumor metabolism is the capability to hijack and remodel existing metabolic pathways to obtain sufficient nutrients from a nutrient-deprived environment and use these nutrients to sustain cell survival and build cellular material (Pavlova and Thompson, 2016). Amino acids serve as the primary compositions of protein, which provide important intermediate substrates for the activation of signaling pathways. Therefore, therapies of amino acid depletion that impair amino acid utilization *via* targeting key enzymes engaged in amino acid metabolism have been extensively studied (Tabe et al., 2019). Arginine is utilized by various metabolic pathways to mediate a series of cellular processes including protein synthesis and production of nitric oxide (NO), creatine phosphate, agmatine, polyamines, ornithine, and citrulline (Tong and Barbul, 2004) (Figure 1). Given that cells can synthesize arginine from citrulline and aspartate *via* argininosuccinate synthase 1 (ASS1) and argininosuccinate lyase (ASL), arginine is considered as a non-essential amino acid (Chen et al., 2021). Thus, arginine depletion may be a promising therapeutic strategy for cancer management.

ASS1 is the enzyme that catalyzes the conversion of nitrogen from ammonia and aspartate from glutamine to form argininosuccinate. The somatic silence of ASS1 expression is commonly observed in a wide range of tumors, such as mesothelioma, non-small-cell lung cancer, myxofibrosarcomas (Huang et al., 2013; Szlosarek et al., 2017; Giatromanolaki et al., 2021). Moreover, low ASS1 expression levels in tumor tissues are associated with unfavorable clinical outcomes in a wide variety of malignancies. ASS1 loss not only confers tumor cells with lack of tumor suppressor functions but also endows tumor cells to be more reliant on arginine supplement. As illustrated in previous review, ASS1 and arginine metabolism represent compelling molecular targets for cancer management. In this review, we summarize the current knowledge of expression pattern of ASS1 and related signaling pathways in cancer and the potential role as a novel therapeutic target in cancer. Besides, we outline how ASS1 affects metabolic regulation and tumor progression and further discuss the role of ASS1 in arginine deprivation therapy. Finally, we review approaches to target ASS1 for cancer therapies.

### ASS1 regulation and signaling networks

ASS1 was initially identified in the liver, and functions as a rate-limiting enzyme for arginine metabolism. Dysregulated promoter methylation is regarded as a key feature of tumors *via* downregulating tumor suppressor genes (Kulis and Esteller, 2010). It is worth noticing that ASS1 silencing is resulted from the epigenetic silencing of the ASS1 promoter *via* methylation of the CpG islands, which has been observed in multiple tumor types (Syed et al., 2013). For instance, ASS1 promoter is frequently hypermethylated in myxofibrosarcoma, resulting in the aberrant loss of ASS1 expression to mediate tumor aggressiveness (Huang et al., 2013). Methylation landscape in cisplatin-resistant bladder cancer has shown that ASS1 is hypermethylated, leading to downregulated expression (Yeon et al., 2018). Aberrant methylation in the promoter of ASS1 makes ovarian tumor cells more resistant to platinum-induced cell death (Nicholson et al., 2009). Tumoral expression levels of ASS1 are also regulated when encountering external factors from the tumor microenvironment to metabolically benefit tumor cell survival. Under acidic and hypoxic conditions, it has been demonstrated that hypoxia inducible factor alpha (HIF1α) binds to ASS1 and downregulates the expression levels of ASS1, providing tumor cells with a metabolic advantage for survival (Silberman et al., 2019). Besides, arginine and glutamine starvation therapies can downregulate HIF1α to upregulate ASS1 expression (Long et al., 2017). Transcription factor c-Myc could directly bind to the promoter of ASS1, mediating arginine deiminase resistance in melanoma cells (Long et al., 2013). Methyltransferase 14 (METTL14), a RNA N6-adenosine methyltransferase, participates in tumor development *via* modulating RNA function (Chen et al., 2020). ASS1 is a target of METTL14-mediated N6-methyladenosine modification (Miao et al., 2022). Specifically, METTL14 upregulation increases mRNA m6A modification of ASS1 and suppresses ASS1 transcriptional expression. Additionally, miRNAs are also essential for the expression of ASS1. In renal cancer cell, miR-34a-5p directly binds to the 3′ untranslated region of ASS1 to reduce its protein expression, while ASS1P3 serves as a competing endogenous RNA for miR-34a-5p to modulate ASS1 expression (Wang et al., 2019). Similarly, lncRNA 00312 attenuates tumor proliferation and invasion by functioning as a competitive endogenous RNA binding to miR34a-5p, making miR34a-5p unable binding to ASS1 to reduce ASS1 expression (Zeng et al., 2020). Considering that post-translational modification is essential for the stability of protein, it mediates diverse cellular processes. For instance, the TRAF2 E3 ubiquitin ligase binds to ASS1, leading to increased ubiquitination and degradation of ASS1 to reduce arginine biosynthesis. The diverse regulation pattern of ASS1 transcriptional and protein expression makes it a promising therapeutic target, and it is necessary to investigate the underlying mechanisms engaged in the expression of ASS1.

### ASS1 and metabolic adaptation

Arginine is a precursor for a wide variety of molecules engaged in the regulation of tumor initiation and development (Zhang et al., 2021). Tumoral downregulation of ASS1 confers tumor cells to be more dependent on extracellular arginine since ASS1-negative cells fail to mediate arginine biosynthesis for tumor survival. The reliance on extracellular arginine has been regarded as arginine auxotrophy, which has been exploited as an’‘Achilles’ heel for cancer management. Thus, ASS1 has been established as a key indicator of arginine auxotrophy. Network analysis of the metabolomics revealed that ASS1-negative glioblastoma cells exhibit altered arginine and citrulline metabolism (Mörén et al., 2018). In ASS1-negative glioblastoma cells, levels of alanine and glutamate are reduced, whereas levels of *α*-ketoglutarate and pyruvate are increased, indicating that ASS1-negative glioblastoma cells are converting less pyruvate to alanine. Multiple pathways for citrulline production are upregulated, and degradation of arginine in ASS negative cells is decreased. In addition, ASS1 functions as an indicator for glutamine-deprivation response. ASS1 inhibition leads to increased sensitivity to both arginine and glutamine deprivation, whereas ASS1 overexpression increases resistance to both arginine and glutamine deprivation (Long et al., 2017). Depletion of extracellular arginine in arginine-auxotrophic cancer cells causes mitochondrial distress and transcriptional reprogramming. Mechanistically, arginine starvation induces asparagine synthetase (ASNS), depleting these cancer cells of aspartate, and disrupting their malate-aspartate shuttle (Cheng et al., 2018). A metabolite profiling of arginine depletion by pharmacological inhibition exhibits elevated serine biosynthesis, glutamine anaplerosis, oxidative phosphorylation, and impaired aerobic glycolysis (Kremer et al., 2017).

Pyrimidines play key role in mediating tumor cell survival and proliferation through providing the nucleic acids and other precursors for cell membrane synthesis (Mollick and Laín, 2020; Siddiqui and Ceppi, 2020). During the synthesis process, the pyrimidine ring structure is formed through a multi-step pathway with glutamine and aspartate as main precursors, which is conversed to dihydroorotate by the three activities of the multifunctional enzyme carbamoyl-phosphate synthase 2, aspartate transcarbamylase, dihydroorotase complex (CAD) (Del Caño-Ochoa et al., 2019; Li et al., 2021). It has been found that tumoral ASS1 expression determines aspartate availability for pyrimidine synthesis (Rabinovich et al., 2015). Intracellular aspartate functions as a substrate for both ASS1 and the enzymatic complex CAD. Tumoral ASS1 loss increases cytosolic aspartate availability for CAD for the synthesis of pyrimidine nucleotides to promote proliferation (Rabinovich et al., 2015). In renal cellular carcinoma, loss of ASS1 and ASL makes aspartate flux towards pyrimidine synthesis to support tumor proliferation (Khare et al., 2021).

The reaction catalyzed by ASS1 is essential for the citrulline-NO cycle. NO is a crucial regulator of multiple cellular processes, such as tumor cell proliferation and angiogenesis (Somasundaram et al., 2019). Decreased levels of NO metabolites and nitric oxide synthase expression have been observed in renal cellular carcinomas and tumor cells lacking ASS1 and ASL (Khare et al., 2021). Combined ASS1 and ASL downregulation significantly reduces aspartate level to impair NO production *via* decreased substrate availability or enzymatic activity. Therefore, ASS1 downregulation influences NO metabolism and promotes tumor cell survival and proliferation *via* mitigation of cytotoxic effects of NO accumulation. Under glucose deprivation, ASS1 expression is induced by c-MYC, therefore promoting tumor cell survival by upregulating NO production and activating the gluconeogenic enzymes *via* S-nitrosylation (Keshet et al., 2020). This metabolic rewiring leads to enhanced gluconeogenesis to increase serine, glycine and purine synthesis. In this ASS1-expressed tumors, purine synthesis inhibition is effective and sensitizes these tumors to immune checkpoint inhibition therapy.

ASS1 loss is associated with polyamine metabolic reprogramming. Results from transcriptomic and metabolomic profiling illustrates that ASS1-lacked cells exhibit reduced accumulation of acetylated polyamine metabolites and therefore a compensatory elevation in the expression of polyamine biosynthetic enzymes (Locke et al., 2016).

### Different role of ASS1 in tumor progression

Numerous studies have shown that tumoral ASS1 functions as a tumor suppressor to sustain the anti-tumor function in a wide variety of tumors (Huang et al., 2013; Allen et al., 2014). Downregulation of ASS1 has been found to play a tumor suppressor role in multiple malignancies. ASS1 exerts its role mainly through its arginine metabolism-dependent mechanisms. It is gradually recognized that ASS1 play different role according to different tumor types. Renal tumors exhibit downregulated ASS1, and loss of ASS1 redirects aspartate towards pyrimidine synthesis and regulates NO production to support enhanced proliferation, uncovering promising metabolic vulnerabilities in renal cellular carcinoma. Besides, it has also been demonstrated that ASS1 may function as a tumor suppressor via metabolism–independent mechanism. In hepatic cell carcinoma cells, ASS1 induces cell death by upregulating ER stress response, independent of arginine metabolism. Specifically, ASS1 overexpression effectively inhibits tumor growth by activating PERK/eIF2α/ATF4/CHOP axis in Huh7 and SNU475 cells, indicating upregulating tumoral ASS1 expression as a promising strategy in tumors with low ASS1 expression (Kim et al., 2021). In renal cellular carcinoma, androgen receptor could reduce ASS1 expression to promote SW-839 and OSRC-3 cell proliferation *via* ASS1P3. Thus, this androgen receptor-induced ASS1 downregulation as a therapeutic target for treatment.

In contrast to its tumor-inhibiting effects, ASS1 has a pro-tumor role in tumor proliferation and metastasis, and the mechanisms seem to vary and differ depending on the specific type of tumor. Based on the differential transcriptional expression analyses of colorectal cancer (CRC) tumors, urea cycle enzymes including ASS1 has been identified to be transcriptionally upregulated in KRAS-mutant primary CRC. Bateman et al. elucidated that ASS1 inhibition impairs CRC survival and proliferation. Metabolomic profiling has pointed that ASS1 inhibition reduces the levels of oncogenic metabolite fumarate, resulting in impaired glycolytic phenotype and reduced CRC progression (Bateman et al., 2017). Snail is a master regulator and transcriptional repressor of epithelial-mesenchymal transition. In colorectal cancer, Snail mediates tumor cell metastasis by preventing non-coding RNA LOC113230-induced degradation of ASS1. Snail regulates arginine biosynthesis by suppressing LOC113230-induced LRPPRC/TRAF2/ASS1 axis (Jia et al., 2021). In gastric cancer, ASS1 knockdown leads to impaired tumor cell invasion by promoting autophagy-lysosome machinery to degrade Snail and Twist (Tsai et al., 2018).

### ASS1 and resistance to chemotherapy

Numerous studies have reported that ASS1 loss is correlated with the development of chemotherapeutic resistance in tumors. For instance, epigenetic silencing of ASS1 confers ovarian tumor cells resistance to platinum chemotherapy. Thus, the expression level of ASS1 has also been regarded as a predictor of clinical outcome in ovarian cancer patients treated with platinum-based chemotherapy (Nicholson et al., 2009). In hepatocellular carcinoma, ASS1 loss has been found to be associated with resistance to cisplatin. Moreover, ASS1 overexpression effectively improves the anti-tumor effect of chemotherapy by activating the PERK/eIF2α/ATF4/CHOP axis. Treatment with decitabine, a hypomethylating agent to increase ASS1 promoter activity, can effectively reduce cisplatin resistance in SNU449 and Huh7 cells. Apoptosis level indicated by cleaved PARP and caspase-3 is significantly upregulated in SNU449 and Huh7 cells after combination treatment with cisplatin and decitabine (Kim et al., 2021).

### ASS1 and arginine deprivation therapy

Tumors with ASS1 loss fail to mediate the arginine biosynthesis, making these cells to be more reliant on extracellular arginine for tumor survival. Thus, arginine depletion therapy may be a promising therapeutic strategy for ASS1-negative tumors. In nearly 70% of tumors, ASS1 loss has been observed, resulting in more efforts to exploit the metabolic vulnerability for development of arginine deprivation therapy. Arginine deprivation therapy is considered to be more effective in ASS1-negative tumors than tumors with low level of ASS1 expression. Arginine deprivation can induce signal alteration. For example, arginine deprivation impairs mTOR and p70S6K activation with consequent inactivation of PI3K/Akt pathway (Wang et al., 2020). PEGylated arginine deiminase (ADI-PEG20) is an arginine-metabolizing enzyme to mediate arginine degradation, which is currently being tested in many clinical trials. ADI-PEG20 has been verified to exhibit anti-tumor effect in a wide range of tumors, including primary acute myeloid leukemia, metastatic melanoma, hepatocellular carcinoma, thoracic cancer (Izzo et al., 2004; Ascierto et al., 2005; Miraki-Moud et al., 2015; Beddowes et al., 2017). ADI-PEG 20 treatment increases T cell infiltration in the low PD-L1 tumor microenvironment to enhance the anti-tumor effect of PD-1 inhibition (Chang et al., 2021). ADI-PEG20 also significantly improves progression-free survival in patients with ASS1-loss mesothelioma (Szlosarek et al., 2017). In pancreatic tumor, ADI-PEG20 can also augment the anti-tumor effect of radiation *via* the activation of ER stress signaling pathway (Singh et al., 2019). ADI-PEG 20 has also been found to be specifically effective in MYC-driven tumors (Chalishazar et al., 2019). ADI-PEG20 can disrupt pyrimidine pools in ASS1-lacked high-grade gliomas to increase tumor sensitivity to the antifolate and pemetrexed (Hall et al., 2019). Although promising results from the preclinical and clinical trials, there are tumors exhibiting resistance to ADI-PEG20. It has been well-established that the re-expression of ASS1 is a main reason for tumor resistance to ADI-PEG20. In mesothelioma cells exhibiting ADI-PEG20 resistance, it is believed that this resistance is induced by regain of ASS1 expression by demethylation of the ASS1 promoter. Considering the efficacy of ADT as a single agent therapy is limited due to frequently observed resistance, combined arginine deprivation and other therapeutic targets may be an answer to improve the therapeutic effect. For instance, HDAC inhibition has been found to induce degradation of a key DNA repair enzyme C-terminal-binding protein interacting protein (CtIP), leading to DNA damage and apoptosis. Arginine deprivation and HDAC inhibition can synergistically mediate DNA damage and degradation of CtIP, resulting in apoptosis (Kim et al., 2020). In addition, a metabolic synthetic lethal strategy has been developed to combine ADI-PEG20 with chloroquine to induce cell death in ASS1-deficient sarcomas (Bean et al., 2016). More importantly, combination treatment of ADI-PEG 20 with chemotherapeutic drugs has been extensively studied. Several clinical trials indicated that this combination treatment has acceptable safety profiles and anti-tumour activity against ASS1-deficient solid tumors (Hall et al., 2019; Szlosarek et al., 2021; Yao et al., 2021; Chan et al., 2022). Summary of ADI-PEG20 utilization in tumor management has been illustrated in Table 1.

**TABLE 1:** Summary of ADI-PEG20 utilization in tumor management.

Tumor types	Regimen	Clinical phase	References
Melanoma	ADI-PEG20	1/2	Ott et al. (2013)
Acute myeloid leukemia	ADI-PEG20	2	Tsai et al. (2017)
Mesothelioma	ADI-PEG20	2	Szlosarek et al. (2017)
Hepatocellular carcinoma	ADI-PEG20	2	Yang et al. (2010)
Hepatocellular carcinoma	ADI-PEG20	2	Glazer et al. (2010)
Hepatocellular carcinoma	ADI-PEG20	3	Abou-Alfa et al. (2018)
Acute myeloid leukemia	ADI-PEG20 + cytarabine	1	
Metastatic melanoma	ADI-PEG20 + cisplatin	1	Yao et al. (2021)
Recurrent high-grade glioma	ADI-PEG20 + pemetrexed + cisplatin	1	Hall et al. (2019)
Metastatic Uveal Melanoma	ADI-PEG20 + pemetrexed + cisplatin	1	Chan et al. (2022)
Non-squamous non-small cell lung cancer	ADI-PEG20 + pemetrexed + cisplatin	1	Szlosarek et al. (2021)
Pancreatic adenocarcinoma	ADI-PEG20 + nab-paclitaxel + gemcitabin	1/1B	Lowery et al. (2017)

The safety and anti-tumor activities of a newly developed arginine depleting drug pegylated recombinant human arginase (PEG-BCT-100) has been verified in patients with advanced arginine auxotrophic tumors (Cheng et al., 2021). PEG-BCT-100 has been tested in chemo naïve post-sorafenib hepatocellular carcinoma, showing well-tolerated with moderate disease control rate (Chan et al., 2021). More clinical studies should be designed to further verify the safety and anti-tumor efficacy, and the potential effects of combination therapy with other chemotherapeutic and targeted agents should also be further explored.

## Implicit of ASS1 in cancer treatment

### Prognostic and predictive value of ASS1 in cancer

In non-small-cell lung cancer, lack of expression of ASS1 in tumor cells is associated with high angiogenesis. Patients with ASS1 expression in tumor cells exhibit a favorable prognosis, which may be related to high density of iNOS-expressing tumor-infiltrating lymphocytes in tumor cells with ASS1 expression (Giatromanolaki et al., 2021). ASS1 is lower in renal cell carcinomas compared with paired normal tissues, and a lower ASS1 expression is correlated with a worse prognosis in patients with renal cell carcinoma (Wang et al., 2019).

### Development of ASS1 activator for cancer treatment

Considering the tumor suppressor role of ASS1 in tumor cells, exploring potent ASS1 agonist to activate ASS1 expression and activity in tumor cells with low ASS1 expression is a promising therapeutic strategy. It has been demonstrated that spinosyn A and its derivative LM-2I exhibit anti-tumor function, which is regulated by activation of ASS1. Mechanistically, spinosyn A binds to ASS1 at the 97th cysteine site in tumor cells, thus increasing ASS1 enzymatic activity and anti-tumor effect (Zou et al., 2021). Currently, the investigation on the development of ASS1 activators is still limited, it is still required to explore deeper to identify the safety and efficacy of ASS1 activators.

## Conclusion

ASS1 is an enzyme that catalyzes the conversion of nitrogen from ammonia and aspartate from glutamine to form argininosuccinate. The down-regulation of ASS1 expression are very common in various tumors, and associated with clinicopathological factors and prognosis. For precision therapeutic purposes, it is important to understand the mechanisms driving malignant diseases to identify the most promising therapy for individual patients. Currently, the down-regulation of ASS1 expression is extensively studied in mesothelioma, non-small-cell lung cancer, myxofibrosarcomas and others. Arginine deprivation leads to immunosuppression, and the removal of L-arginine can improve the elimination of arginine-auxotrophic tumors. However, the removal of L-arginine has no inhibition on the development of non-auxotrophic tumors, given that these tumors synthesize L-arginine from citrulline by expressing ASS1. Therefore, it is necessary to identify the auxotrophic and non-auxotrophic tumors. To date, numerous studies of phase 1–3 clinic trials are being conducted to evaluate the efficacy of ADI-PEG20 therapy in diverse tumor types for its anti-tumor activity, it is still required to explore deeper to identify the cancer types that can be effectively treated with ADI-PEG20 therapy. In addition, combination of ASS1 activators with anti-tumor drugs like chemotherapy and TKIs could augment the anti-tumor effect of traditional regimens. Given that ASS1 is commonly downregulated in multiple tumor types and participates in the regulation of tumor development depending on diverse mechanisms, it may be a robust potential therapeutic target for cancer management.
